# Mobile phone applications and their use in the self-management of Type 2 Diabetes Mellitus: a qualitative study among app users and non-app users

**DOI:** 10.1186/s13098-019-0480-4

**Published:** 2019-10-16

**Authors:** Bronte Jeffrey, Melina Bagala, Ashley Creighton, Tayla Leavey, Sarah Nicholls, Crystal Wood, Jo Longman, Jane Barker, Sabrina Pit

**Affiliations:** 1 Western Sydney University, Sydney, Australia; 2 University of Sydney, University Centre for Rural Health, Sydney, Australia; 3 Western Sydney University, University Centre for Rural Health, 61 Uralba Street, Lismore, NSW 2480 Australia; 4 School of Medicine, University of Sydney, Sydney, Australia

**Keywords:** Type two diabetes mellitus, Mobile phone apps, Self-management, Smart phone, mhealth, ehealth, Digital technology, User experience

## Abstract

**Background:**

Mobile phone applications (apps) have been shown to successfully facilitate the self-management of chronic disease. This study aims to evaluate firstly the experiences, barriers and facilitators to app usage among people with Type 2 Diabetes Mellitus (T2DM) and secondly determine recommendations to improve usage of diabetes apps.

**Methods:**

Participants were aged ≥ 18 years with a diagnosis of T2DM for ≥ 6 months. Semi-structured phone-interviews were conducted with 16 app and 14 non-app users. Interviews were based on the Technology Acceptance Model, Health Information Technology Acceptance Model (HITAM) and the Mobile Application Rating Scale. Data were analysed using deductive content analysis.

**Results:**

Most app-users found apps improved their T2DM self-management and health. The recommendation of apps by health professionals, as well as positive interactions with them, improved satisfaction; however, only a minority of patients had practitioners involved in their app use. All non-app users had never had the concept discussed with them by a health professional. Facilitators to app use included the visual representation of trends, intuitive navigation and convenience (for example, discretion and portability). Barriers to app use were participant’s lack of knowledge and awareness of apps as healthcare tools, perceptions of disease severity, technological and health literacy or practical limitations such as rural connectivity. Factors contributing to app use were classified into a framework based on the Health Belief Model and HITAM. Recommendations for future app design centred on educational features, which were currently lacking (e.g. diabetes complications, including organ damage and hypoglycaemic episodes), monitoring and tracking features (e.g. blood glucose level monitoring with trends and dynamic tips and comorbidities) and nutritional features (e.g. carbohydrate counters). Medication reminders were not used by participants. Lastly, participants felt that receiving weekly text-messaging relating to their self-management would be appropriate.

**Conclusions:**

The incorporation of user-centred features, which engage T2DM consumers in self-management tasks, can improve health outcomes. The findings may guide app developers and entrepreneurs in improving app design and usability. Given self-management is a significant factor in glycaemic control, these findings are significant for GPs, nurse practitioners and allied health professionals who may integrate apps into a holistic management plan which considers strategies outside the clinical environment.

## Background

In Australia, people living in regional or remote areas have higher rates of diabetes and experience worse health related outcomes than people living in urban areas [1]. Type 2 Diabetes Mellitus (T2DM) is a major contributor to higher death rates outside major cities and accounts for 6% of excess deaths in all age groups [1, 2]. This is attributed to several factors, including decreased accessibility to health services (fewer health professionals and decreased financial accessibility), decreased testing for diabetes and possibly less effective management [2]. Facilitation of self-management strategies may help to overcome these issues.

Self-management is considered the most important factor in ensuring well-controlled blood glucose levels (BGL) and, thereby, preventing diabetes complications [3, 4]. It has the potential to ease the burden on the healthcare system by encouraging patient autonomy and allowing disease monitoring outside clinical settings [5–8]. Self-management strategies include tracking blood glucose trends, adhering to medication or insulin therapy, monitoring nutrition and increasing physical activity [9]. Current research has established that apps are feasible tools to improve self-management of diabetes [4, 6, 10]. App use has been demonstrated to result in positive self-management behaviours, such as improved diets and attitudes towards diabetes self-management, increased physical activity and BGL monitoring [4, 11]. Furthermore, a recent meta-analysis has demonstrated that among people with T2DM, the use of diabetes apps as an adjuvant to standard self-management results in a clinically significant reduction in HBA1C, a long-term marker of BSL control [6, 8].

Despite these positive outcomes, in Australia, only 8% of people with T2DM are reported to use apps to support diabetes self-management [12]. This poor uptake is multifactorial, with limitations including a lack of education integration into app technology, generic and impersonal information, perceived difficulty of use and an inability to export data or integrate with health professionals’ records [4, 7, 9, 13]. Additionally, there is concern about the feasibility of sustained use of apps [14–16] with minimal data exploring long term app usage outside of short randomised control trials. From the patient perspective, studies have identified that people with T2DM do not believe apps will be useful, resulting in low uptake [12, 16–18]. Recent data from an Australian qualitative study demonstrated that people with T2DM would prefer an app to address the practical aspects of diabetes self-management and to improve, and reduce the cognitive burden of self-management [17]. Further studies using focus groups for app development have highlighted the importance of blood glucose monitoring, dietary tracking, education, interactive content, peer support and realistic goal setting [19–22]. Despite this, the uptake of apps usage to support diabetes self-management remains low, [12]. Additionally, current research has concluded that there is a paucity of qualitative data on current user app experience and factors influencing consumer engagement [5, 11, 12, 18].

The lack of qualitative evidence surrounding health app usage was addressed by Anderson et al. [5] who conducted the first study combining three theoretical frameworks to qualitatively explore users’ experience of apps in relation to chronic conditions; The Technology Acceptance Model (TAM) measures how users accept technology and is based on the Theory of Reasoned Action [23]. The Health Information Technology Acceptance Model (HITAM) furthers the concepts in TAM to focus on health by incorporating the Health Belief Model [24]. The Mobile Application Rating Scale (MARS) includes theoretical constructs of engagement, functionality, aesthetics and information quality [25]. The integration of these frameworks provides robust theoretical grounding for research into the consumer experience of mobile phone apps [5].

The present study uses the interview guide developed by Anderson et al. [5], based on the three frameworks, in relation to T2DM. To our knowledge, there are no studies that have focused on app use in an Australian rural population where issues of healthcare access may increase the importance of self-management strategies.

Overall, further qualitative evidence is required to obtain an accurate summary of consumer experiences and preferences to shape targeted app innovation and development. User-centred diabetes apps have the potential to improve health outcomes, particularly in rural areas where access to formal health services is relatively restricted. Therefore, this study aims to acquire a greater understanding of the perceived useful features, facilitators and barriers to app usage for the self-management of T2DM in a rural population.

## Method

### Participants

Participants were recruited through responding to a flyer. These were distributed amongst general practices, allied health clinics, Facebook groups and pages which were specific to either diabetes or rural communities, and diabetes support groups. Participants were also recruited through snowballing techniques, whereby participants already in the study recruited future participants by informing people in their social network about the study [26]. These participants contacted researchers to express interest in taking part in the study. The inclusion criteria were: participants aged over 18 years from rural locations in Australia (RA2 or above), with a self-reported T2DM diagnosis for greater than 6 months, and smartphone ownership. Defined by the Australian Government Department of Health, RA2 or above is any area outside of major cities, including inner (RA2) and outer regional (RA3), remote (RA4) and very remote locations (RA5) in Australia. In this classification, remoteness is determined according to population and distance to services [27]. Participants were separated into app and non-app users. All health apps which could be used to facilitate diabetes self-management behaviours were accepted, including diabetes specific participants ranged in age, sex, rurality, app use experience, distance to GP and endocrinologist (time to reach measured in minutes) and diabetes management (management strategies identified by participant) (Table 1).

**Table 1:** Summary of participant characteristics

Patient characteristic	N (%)
App use	
Current or prior	17 (57%)
Never	13 (43%)
Age	
30–39	1 (3%)
40–49	7 (23%)
50–59	5 (17%)
60–69	12 (40%)
70–79	5 (17%)
Gender	
Female	14 (47%)
Male	16 (53%)
Rural classification	
RA2	18 (60%)
RA3	7 (23%)
RA4	5 (17%)
Distance to GP (mins)	
0–30	22 (74%)
31–60	6 (20%)
61–90	1 (3%)
301–360	1 (3%)
Distance to endocrinologist (mins)	
0–60	6 (20%)
61–120	1 (3%)
181–240	2 (6%)
301–360	2 (6%)
NA	19 (63%)
Diabetes management	
Lifestyle modifications	6 (20%)
Medication	11 (37%)
Medication and insulin	7 (23%)
Insulin	4 (13%)
NA	2 (6%)

Diabetes was not managed by an endocrinologist or chose not to state management

*NA* not applicable

### Interview guide

Semi-structured interview guides were developed for app and non-app users (Appendix: Tables 5 and 6) adapted from Anderson et al. [5]. Briefly, the following constructs used by Anderson et al. were used in this study: ‘perceived ease of use’ and ‘perceived usefulness’ from TAM, personal and social factors (self-reflection, motivation and recommendations) from HITAM and aesthetics (font size, text and dialogue boxes) from MARS. Any constructs that were duplicated across the three frameworks were included once only by Anderson et al. [5]. Additional questions were added to explore factors related to mobile phone acceptance and health app usage [5]. Upon review of the Scheibe et al. [28] study from which the supplementary questions had been derived, an additional question was added to the non-app user guide: “What features would you want in the app to make it useful for you?” [5]. Asking this question allowed the guide to gain more comprehensive insight into the features of a useful diabetes app [28]. It is important to get multiple perspectives from different types of people including non-users to allow improvement of future versions of diabetes apps. People who do not use diabetes apps may have preferences or perspectives about diabetes apps that diabetes app users may not think of. Additionally, people who are currently not using diabetes apps may well be using other apps for other purposes so could translate their experiences to diabetes specific apps. Subsequently, a pilot test was undertaken by the researchers. This revealed that, whilst all questions were necessary, the flow was poor in an interview setting. Minor adjustments were made to the order of questions to facilitate a more conversational tone.

### Data collection

Participants were sent an information sheet prior to providing verbal consent. Interviews were audio-recorded and a reflective journal was written immediately following each interview using a previously published format [29]. The reflective journal helped to capture practical details of the interview and to assist with recollection subsequently of initial impressions, key issues and ideas of interviewers which were used in discussion with the rest of the interview team as part of developing a growing understanding of the data across the team. The demographics of the participants, including age, gender, education level, occupation, location, distance from general practitioner (GP) and endocrinologist, and diabetes management were collected. Information on features included in apps used by participants was also recorded including: exercise tracking, timely medication administration, BGL, diet monitoring and suggestions, self-management education, weight management, blood pressure monitoring and patient monitoring by clinicians (Appendix: Table 4) [4].

### Data analysis

Interviews were transcribed verbatim by the person who had conducted the interview (BJ, MB, AC, TL, SN and CW) and accuracy checked against the audio recordings by a different researcher (AC, TL and SN). In this way, all researchers became familiar with all interviews. All interviews were coded using NVivO 11.0 [30]. Data were analysed using deductive content analysis following Elo and Kyngas [31], with initial broad categories based on each of the constructs in the MARS, TAM and HITAM. An initial structured analysis matrix was developed from these frameworks. This initial matrix was then trialled and refined against three different transcripts from the actual respondents (two app and one non-app user) and in response to a review of the notes from the reflective journal. Subsequently, a number of more nuanced sub-categories were added into the matrix (Researchers: BJ, MB, AC, TL, SN and CW). The matrix was again trialled and refined on a further four transcripts from the actual respondents (two app and two non-app users) (Researchers: AC, TL and SN)., allowing for continuous discussion and reflection until a matrix was finalised. Two authors then independently coded one transcript and coding was concordance-tested in NVivO, showing good agreement (Researchers AC and TL). A final codebook was agreed and the remaining interviews were coded by pairs between researchers AC, TL and SN. During the analysis, data categorised within constructs were scrutinised to identify commonalities and differences in views and experiences across the range of participants. To ensure data saturation, data was collected until no new information was elicited, at which point (after 28 interviews) two further interviews (one app and one non-app user) were undertaken. Coding these final two interviews confirmed saturation (Researchers: AC, TL and SN).

## Results

Thirty participants were recruited: 20 from Facebook (including rural ‘buy/swap/sell’ and ‘community noticeboard’ groups and the Diabetes Australia Facebook page), eight from snowballing techniques and two from diabetes support groups. Interviews lasted between 25 and 45 min and were conducted between September 2017 and February 2018 with 17 current or prior app users and 13 people who had never used an app (Table 1). There were 14 female participants (47%) and 16 male participants (53%). Ages ranged between 30 and 79 years, with the most common age bracket being 60–69 (40%). Eighteen were from inner regional areas (RA2), 7 from outer regional areas (RA3) and 5 from remote areas (RA4).

None of the apps used by participants included all self-management tasks listed by El-Gayer et al. [6]: frequent BSL monitoring, suitable diet, physical exercise, timely medications dosage, blood pressure monitoring, weight management and self-management education (Appendix: Table 4). The most comprehensive apps were Diabetes Journal and Accu-Chek. The most supported self-management tasks were diet and exercise monitoring.

**Fig. 1:**
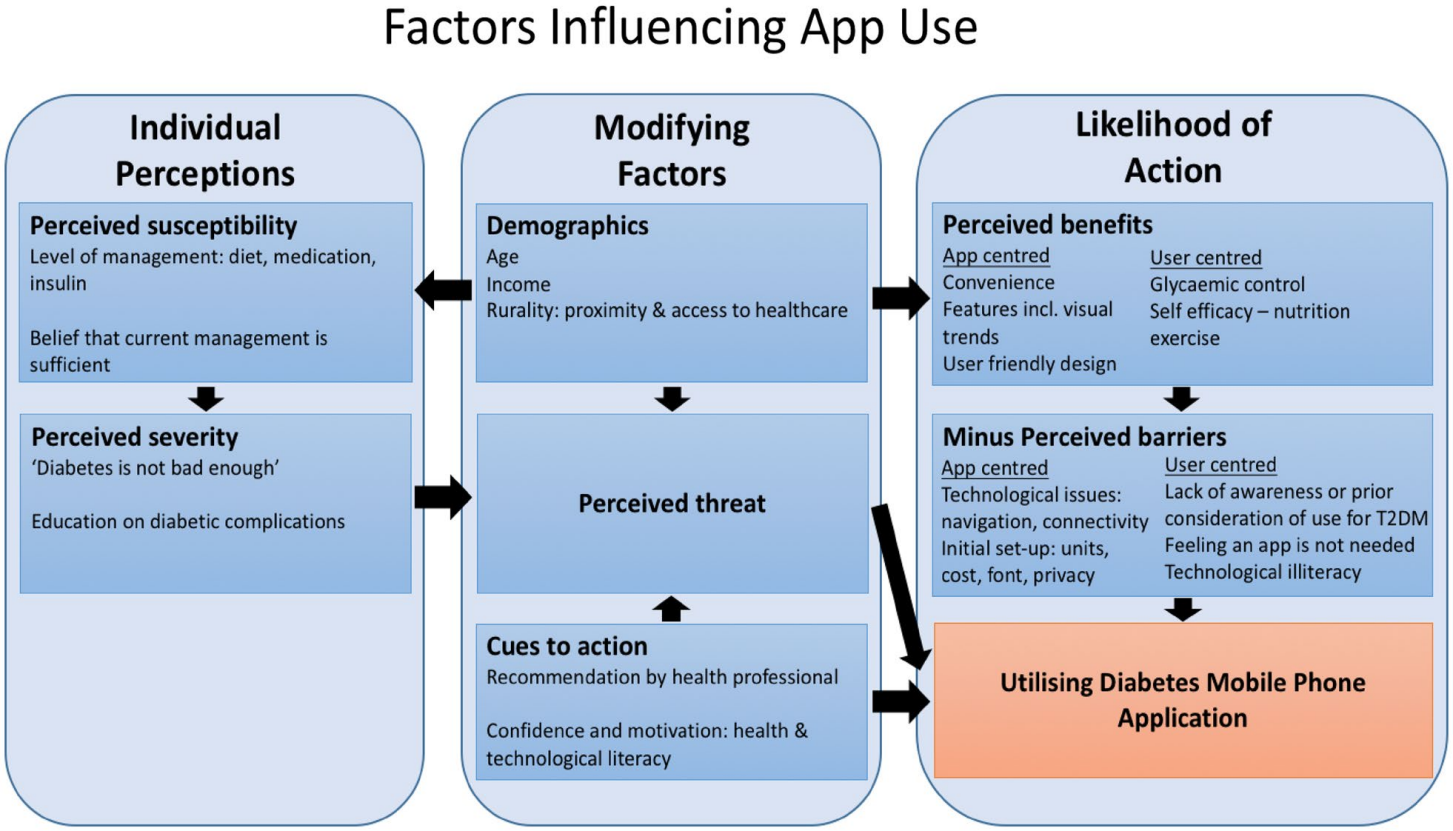
Factors which contribute to the likelihood of app use modified from the Health Belief Model and HITAM [24, 32]

Table 2 shows the summary of key findings of barriers and facilitators of app usage and useful features of app use. Factors influencing app use are summarised in Fig. 1 and are based on a modified version from the Health Belief Model and HITAM.

**Table 2:** Key findings of barriers and facilitators of app usage and useful features of app use

Barriers to using apps
App-specific
Technological issues: app failing to work as intended (e.g. connectivity ongoing issue), not being user friendly, difficult to navigate
Initial setup issues: units of measurement (American vs Australian), cost of app, font size
User-specific
Perceptions of app use:
Feeling they did not need an app
Not knowing about available apps
Not having thought of using an app for self management before
Self-perception of diabetes
“Not being bad enough”
Current care being sufficient
Self-perception of technological literacy
Internet connectivity

Perceived facilitators and useful features of app use
Apps perceived to be useful and majority would recommend the app
App specific
App user-friendly: easy navigation, clear designs, intuitive technology
Convenience: ease of blood glucose monitoring, discretion of using phone, inbuilt exercise technology, time taken to perform tasks
Features of apps: BGL connectivity with glucometer, calculating content of food
User-specific
Personal and social factors
Health literacy and technical literacy likely to influence positive attitudes towards app use
Many open to the idea of using an app; however, some felt current management was sufficient
Interaction with health-care professionals
Recommendation by healthcare profession well received. Others stated they would use an app if their GP would recommend it
Use of app not often disclosed to health professional
Healthcare professional discussing app use encouraged self-reflection on diabetes management

### 1. Barriers to using an app

App-specific barriers were defined as issues app users had when using apps, discouraging them from further use. User-specific barriers were defined as factors inherent to the user, encountered both in app and non-app users.

#### 1.1 App-specific

Technological issues were the most common problem of the app, and included technology failing, not being user-friendly or difficult to navigate. Issues with technology failure included connectivity, such as Bluetooth connection, and the app crashing. Some participants faced this as an ongoing issue.

*“…it has failed four times [the app]. It has been working, and now suddenly I lose the link. So I’ve had to re*-*establish that link with my smartphone, even though the smartphone is sitting right next to it… I’ve finally given up.” (Participant 4, 65* *year old male, app user)*

Some participants had issues with units of measurement, which were American, and could not be adjusted to an Australian measurement standard.

*“a lot of them were American made so therefore their blood glucose measurements are different to ours… they also didn’t have an option to change the blood to our readings…it was just I found it was a headache.” (Participant 21, 45* *year old female, app user)*

Other participants found the app navigation cumbersome and unintuitive, with too many steps to perform tasks and complicated layouts.

*“To me it just wasn’t user friendly… Hard to navigate, that’s the only words I can give you. And tedious, it seemed to be tedious.” (Participant 14, 63* *year old female, app user)*

Multiple participants spontaneously discussed the cost of the app. For one participant this was a significant issue, which they believed was one of health equity, relating back to user-specific issues of affordability.

*“It’s a medical issue. They should be free really, to access full features and everything else… you know, it can be life and death. If someone has a smart phone they can have an app, but they can’t access it like I said because they can’t afford to.”* (*Participant 21, 45* *year old female, app user*)

One participant reported that small font size was a significant barrier to app use and consequently favoured other methods of self-management, such as printing information with larger font size, diabetes magazines and journals.

#### 1.2 User-specific

There were three main barriers amongst non-app users: feeling they didn’t need an app, not knowing about available apps, not having previously considered the use of apps for self-management. Firstly, many participants (almost exclusively non-app users) stated they did not need an app to manage their diabetes. Participants often described their diabetes as ‘not bad enough’ to need an app, or thought that their current management was sufficient and wouldn’t be improved by an app.

*“Researcher: Would you ever consider using a mobile phone app to help manage your diabetes?*

*Participant: probably not, no I’m, I think I’m keeping it well under control…taking the bloods every day, and, following you know what the doctor says.” (Participant 29, 58* *year old male, non*-*app user)*

Secondly, many participants were not aware of apps or their features and often struggled to answer questions specifically relating to app features without prompting. Thirdly, many participants stated they had simply never thought of using an app to manage their diabetes, despite using other apps on their phones. Some non-app users considered management the domain of their GPs and did not see the need for app use unless specifically asked by their GP to do so.

*“…my GP and all that, I’m extremely confident in them, they haven’t mentioned it to me at this stage… I would be quite happy to move to an app if my GP agrees to that.” (Participant 28, 70* *year old male, non*-*app user)*

Further user-specific barriers were also identified across app users and non app users. Several participants self-identified as having poor technological literacy. This had some overlap with participants who had negative attitudes towards technology. Some attributed their lack of technological literacy to their age. Participants expressed difficulty with newer technologies, including not knowing how to download an app, and frequently a lack of desire to learn these newer technologies.

*“Researcher: Do you know why you wouldn’t use those bits [features of the app]?*

*Participant: Probably ignorance or my age probably has a factor in that…that’s an older person’s thinking, you know, I won’t fiddle because it might bite me.” (Participant 19, 49* *year old female, app user)*

*“I wouldn’t know how to download an app to be quite honest with you.” (Participant 2, 69* *year old male, non*-*app user)*

Lastly, internet connection was an issue noted for some participants due to their rural location.

*“I do have trouble setting up my phone to get apps because my internet service is not very strong here. To use my computer, I have to hotspot and sometimes that’s just, there’s just no coverage… I think it’s just the lack of service.” (Participant 23, 69* *year old female, app user)*

In addition to these user specific barriers amongst non app users, lack of health care professional recommendations was a barrier identified by app users and non app users. Most participants had not received a recommendation to use an app from a healthcare professional, nor had they told their GP they were using an app. Reasons for this included thinking their GP would not have time or an interest in addressing app use, or having an older GP whom they assumed was unfamiliar with the technology.

*“No, not at all… when I was using the app I had an elderly gentlemen [GP] and so he wouldn’t have been interested in that sort of thing.” (Participant 14, 63* *year old female, app user)*

Participants who had spoken to their GP had positive experiences, indicating that their GP found the app helpful, and worked alongside the participant to use the app for management. One participant said their GP has since recommended their app to other patients.

*“Oh, he sits there and looks at it and we look at each other and we say yep, everything’s fine. But no… he scans them and files them on my record so it must be of some use to him… I’m telling him that I’m feeling OK and everything’s fine and I guess it reinforces that. He can’t argue with a graph.” (Participant 26, 74* *year old male, app user)*

### 2. Facilitators to using an app

#### 2.1 App-specific

The majority of app users found their apps very user-friendly. They described simple and straightforward navigation, clear layouts and designs and intuitive technology. Many participants also identified convenience as something they liked in their app. What this meant varied from app to app and included: being able to measure BGL easily and discreetly while away from home, being able to carry your phone and thus the app with you, or having the app count steps automatically.

*“Sometimes it’s actually just easier to smartphone it than it is to find your book and write it down and fiddle around like that, you just tap it in.” (Participant 21, 45* *year old female, app user)*

Most of the participants reported that their technology worked well without any significant issues. They also were happy with the time taken to perform tasks and thought they worked quickly and efficiently.

Visual representations of trends, particularly graphs of BGLs, were highly valued and were a significant reason for continuation of app use.

*“It produces an average for the beginning period which is customisable, so I can go back three months, 6* *months, whatever, so you can immediately spot any trend, the exercise level and the carbohydrates graph, they’re bar charts and very easy to follow… You can see the pattern all together so I find it very, very useful.” (Participant 26, 74* *year old male, app user)*

#### 2.2 User-specific

User-specific facilitators were often the counterparts to barriers experienced by some participants.

Many app users self-identified as having good technological literacy, which meant they found it easier to use apps. Some participants also had positive attitudes to technology, which meant they were more likely to consider and continue using apps. Only two participants had been recommended an app by a healthcare professional, neither of whom were GPs. Recommendations are significant as participants may not have otherwise known about apps.

### 3. Experiences of using apps

#### 3.1 Tracking and monitoring

A commonly used feature was recording BGL and utilising associated graphs showing trends. This was aided in apps which had glucometer to app connections and allowed direct transmission of data from the glucometer. Tracking BGL measurements was associated with daily use of the app, thus increasing engagement. Other apps had features that encouraged and tracked exercise or nutritional monitoring.

*“But um with this it’s actually averaging it all out. And I really like that side of things, it helps me keep at it.” (Participant 30, 60* *year old male, app user)*

#### 3.2 Education

As a first line educational resource, most participants used Google. This was the most easily accessible source. Issues noted with this source were the difficulty of assessing the reliability of information and the lack of personalised information. Most participants liked accessing information from healthcare professionals, usually their GP. Participants perceived this information to be reliable and personalised. Other participants read articles, magazines or pamphlets as their most utilised form of education. Another form of education participants liked was face-to-face communication and/or peer education, including support groups, informal chatting with friends diagnosed with diabetes and phone-based services. This was generally perceived as reliable and personalised information.

#### 3.3 Personal and social factors

In terms of self-management, participants struggled the most with regular BGL monitoring and meeting target levels, weight management and diet, despite these factors being available for tracking and monitoring.

Some participants also mentioned factors that were specific to living rurally and indicative of health inequity. These included GP accessibility, a reduction of services in the area and poor phone and internet service.

*“There used to be a Diabetic Association office here in (RA 2)… But with the changes, where the pharmacist took that over, those offices were closed… they used to provide free sessions on a variety of things like diet, or managing diabetes, or testing your equipment, and I used to go to those quite regularly, they were very useful. And you know, there was talking, swapping of ideas, here’s the latest trends, here’s the latest equipment. It was really, really useful. But since, you know, that’s been a loss, a big loss, where I think a lot of people, particularly in regional areas.” (Participant 4, 65* *year old male, app user)*

### 4. Perceptions of usefulness

App users generally perceived their apps to be useful. Additionally, most people who were asked would recommend their app to others. Only one participant would not recommend their app.

#### 4.1 Did not meet participant needs

Three of seventeen app users said their app did not meet their needs. Of these participants, two thought the app did not provide anything superior to what they could do themselves without an app, for example, writing their BGL down physically.

One participant specifically wanted trends and averages and found her app did not meet that need.

*“…what I wanted was like, a reading for the day, like a total reading and, and how much insulin I’d had each day and then sort of to see over a month what my average reading was…” (Participant 23, 69* *year old female, app user)*

#### 4.2 Impact on management

Ten app users talked about how the app had improved their diabetes management. Of those who thought it had not, many referred to their apps as tools to help them with certain aspects of their diabetes, but believed that improved management overall was ultimately their responsibility with help from the GP. Those who did notice an improvement in management felt it could be attributed to a particular app feature. These included being able to associate trends in BGL with foods they had eaten, being able to calculate the carbohydrate content of food, and having BGL measurements all stored in one place.

#### 4.3 Non-app users

Many non-app users were responsive to the idea of app use, saying they were open to trying it or would consider using one in the future, especially if recommended by a GP.

*“Researcher: Would you ever consider using a mobile phone app to help manage your diabetes?*

*Participant: Uh, yes, if it needs managing. I don’t know I feel fine.” (Participant 12, 68* *year old male, non*-*app user)*

### 5. Recommendations

Table 3 summarises features participants would like in an app and are broadly placed into five categories: educational features, monitoring and tracking of health information features, nutritional features, medication reminders, text messaging. Quotes are added to support the findings.

**Table 3:** Recommendations

Features	Items	Quotes
Educational features	Educational component as part of the app: preferred topics were related to diabetes complications including: end organ damage (e.g. nephropathy, stroke, myocardial infarction) and hypoglycaemic episodes Nil interest in in-app educational features and preference of information from other sources	*Researcher: “What diabetes issues do you think are important for people to have information on?* *Participant: “Um, the diabolical effect that diabetes has on your body…Effect on …you know your organs. (Participant 10, 68* *year old female, non*-*app user)*
Features that include monitoring and tracking health information	BGL monitoring with trends paired with dynamic tips Additional self-management tasks: blood pressure monitoring, weight monitoring and activity tracking Reminders for exercise and appointments	*“I’ve got a useless memory and I can’t remember. I wouldn’t be able to remember *< *BGL *>* what the um… If I did happen to check multiple times a day, I wouldn’t remember what they were anyway. So I wouldn’t be able to give an average or trend or anything like that. If I had to do it off memory. “(Participant 27, 37* *year old male, app user)*
Nutritional features	Carbohydrate calculators, diabetes specific recipes or meal suggestions Diabetes friendly food suggestions, an app that says if a food is/is not suitable for people with diabetes	*Researcher:”… and if you were to use an app, what features would you want in the app to make it useful to you?” Participant:” um, the biggest issue is trying to um, decide with a decent menu… yeah. So much stuff out there’s got sugar in it and you’ve got to try to avoid it you know.” (Participant 11, 48* *year old male, non*-*app*-*user).*
Medication reminders	Content with their own medication routine Medication features, such as an app that allows a medication list to be uploaded or reminds one when it is time to collect a new script (such an app exists and is used by the participant who brought this up)	*“I think a feature such as being alerted about your medication, I think could be highly useful. I mean I get messages about these things I have to go to, so that was highly useful.” (Participant 4, 65* *year old male, app user)*
Text messaging	Weekly text messaging would be an appropriate time frame	*“You get sick of seeing it. But if it was weekly one or a fortnightly one or something, then I’d be more likely to read it because I’m not just going to flick it off and get rid of it, so.” (Participant 9, 44* *year old male, app user)*

## Discussion

This study examined the attitudes of people with T2DM regarding their experience, perceived useful features, facilitators and barriers to the use of mobile phone applications for self-management as elicited by the theoretical frameworks of TAM, HITAM and MARS [23–25]. Mobile phone applications can improve T2DM self-management. Overall, the results demonstrate the potential of apps to improve self-management and perceptions of self-efficacy. Useful features reported included visual representation of health trends (i.e. BGL graphs), convenience including the discretion and portability of mobile phones, and user-friendly functions and designs. The most notable barriers to app use were a general lack of awareness of apps as potential healthcare tools (that is non-user participants had never considered them before), inadequate internet access in rural areas, perception of their current T2DM management and severity, costs and technological literacy. Significantly, very few participants were recommended or encouraged to use an app by their healthcare professionals; however, participants who interacted with their healthcare professional around an app found this useful. These results have important implications for clinical practice and future application design.

### Perceived usefulness and facilitators

In general, participants perceived their applications to be useful, with a majority of participants concluding app use improved their diabetes management. This may be attributable to findings by El-Gayer et al. [4] who reported app use was associated with improved attitudes toward diabetes self-management. A reported useful app feature was the visual representation of trends in the form of graphs and averages, with participants describing these trends as a source of motivation. This is consistent with findings from Anderson et al. [5], which suggest motivation to sustain app usage is dependent on the inclusion of features with high quality aesthetics, functionality and user engagement. A recent qualitative study suggests that data tracking and visualisation allows users to gain understanding of how BGLs interact with other factors [17]. Significantly, this provides an advantage over traditional forms of recording BGL readings, particularly as increased awareness of BGL has previously been proposed as a mechanism by which apps improve HBA1C levels of users [33, 34]. Notably, the incorporation of BGL tracking was linked by participants to their daily app use in the present study. This is significant as frequent self-monitoring is known to improve glycaemic control [3, 4], although this effect may be limited in individuals with T2DM who are not insulin dependent [35]. Another major perceived advantage, when compared to traditional forms of monitoring health, included the discretion of using a mobile phone as well as the constant ability to monitor and record due to portability. Consistent with the findings of Brzan et al. [9], participants also reported their apps to be useful for self-management tasks, including monitoring nutrition and increasing physical activity.

An important potential facilitator was the app being recommended by a healthcare professional. Whilst only two participants in this study were recommended the app by their healthcare professional, these participants found that using it in conjunction with their GP facilitated improved self-reported app satisfaction and progress. Data from a recent meta-analysis demonstrates that healthcare professional feedback augments the decrease in HbA1c associated with diabetes app use [6], reiterating the importance of health care professional involvement. Another systematic review suggests that apps that include tools to remotely communicate with health professionals or apps used in association with frequent healthcare visits have improved benefits on HbA1c [36]. Our study expands on this information and highlights that people with T2DM would be willing to engage in self-management tasks facilitated by an app if recommended by or used in conjunction with their healthcare team. Despite this, many professionals remain cautious and a previous study identified that over one-third of health professionals would like guidance on app recommendations [37]. Diabetes applications that enable data exporting provide a mechanism of incorporating the health professional in the person with diabetes self-management [9]. Developing evidence-based apps, which clearly adhere to up-to-date guidelines, is a priority to engage clinicians in recommending diabetes health apps in a currently unregulated market [38, 39].

### Barriers

Barriers to engaging with diabetes apps encountered by participants were related to either technical issues or user perception of app use. Technical issues related to poor app design: the app failing to work as intended, not being user friendly and difficult to navigate. Perceived ease of use of health technology, a concept explored in the TAM and HITAM (Fig. 1), alters self-efficacy and, therefore, these technical barriers can serve to decrease intention to use the app [23, 24]. Brzan et al. [9] propose face-to-face training in app use to overcome this barrier. Compounding the technical issues, was perceived technological illiteracy, a concern more prevalent in older respondents. Older people have reported increased difficulty navigating and engaging with diabetes apps [28] and in comparison to younger age groups have been shown to be less likely to benefit from diabetes app use [6]; however, as younger individuals are familiarised with the use of technology and apps for health purposes, their incorporation into management of T2DM is rendered more valuable in the future, particularly in Australia’s ageing population [12, 13, 36]. Furthermore, the age of diagnosis of T2DM is decreasing, which again increases the technology literate audience for health apps [40].

A technical barrier explored, which was specific to rural populations, was data connectivity. Participants noted inability to use some app features when out of range of data signals. Developing “off-line” features, in future applications, could further engage rural populations. Furthermore, app users also noted issues with glucose units being presented in mg/dL compared to mmol/L, which is the predominant unit used in Australia. Therefore, app design should include units for glucose and nutritional intake which are interchangeable or that comply with current guidelines utilised by health professionals in their respective countries to simplify and propagate use. Minimising these technical issues is essential as frustration with app technology is recognised to lead to loss of motivation and dropout for people with T2DM [33, 41].

Three main user-specific barriers which prevented participants using apps were identified: feeling they did not need an app, a lack of knowledge of available apps and not having previously considered an app for self-management. Those who felt they did not require an app also held the perception that their diabetes is “not bad enough” or that their current care is sufficient. Similar results were described in the MILES-2 study in which the main identified barrier to app use was the perception apps would not help diabetes management [12]. Desveaux [33] further demonstrates that an individual’s beliefs about apps not only impacts on engagement, but also clinical outcomes (including HBA1C). This is consistent with the Health Belief Model (Fig. 1), on which HITAM is based, whereby a decreased belief in personal threat, together with decreased belief in the effectiveness of a proposed behaviour, predicts the likelihood of engaging with that behaviour [32]. There is likely, however, an incongruence with perceived and actual “seriousness” of diabetes as the literature suggests rural populations are particularly vulnerable to poor outcomes of T2DM [1, 2].

Lack of knowledge around applications was apparent in the majority of interviews with non-app users. All the non-app users in our study had never been introduced to the concept of apps by their health professionals and many also commented on the trust and value they placed on their professionals’ opinions. Previous studies have also emphasised that lack of patient–provider interactions as a barrier to engaging with diabetes apps [33, 41, 42]. This reiterates that healthcare professionals should have a role in introducing reliable and effective apps. As previously mentioned, this would require an enhanced awareness of the benefits and formulation of guidelines for the recommendation of reputable and verified applications.

### Recommendations

Key recommendations from the present study for future app development centred on improving the education provided by apps and increasing customisation features. Participants described a self-perceived difficulty in accessing reliable and personalised diabetes information, with most utilising multiple information modalities. The majority of apps used by participants did not provide any educational information and those that did provided generic information (Appendix: Table 4). This is reflective of the overall market of available diabetes self-management applications, which currently fail to integrate educational information [4]. This is particularly surprising as both clinical guidelines and the literature emphasise the role of education in improving motivation for self-management and behaviour change [4, 43]. Interestingly, most participants were enthusiastic about the inclusion of information in diabetes apps, with preferred topics revolving around complications, such as end organ damage and hypoglycaemic episodes. This correlates with the literature which states that education may increase competence and reduce fear surrounding these situations [36]. Furthermore, the areas of management which participants identified as struggling with most, glycaemic control, nutrition and weight management, can be addressed with education provided in apps; however, the effectiveness of remote education alone without a face-to-face component is not yet robustly determined in the literature [44]. Respondents suggested that features such as carbohydrate calculators, diabetes specific recipes or meal suggestions could be incorporated into apps to improve their self-reported management deficits. Respondents also suggested incorporating dynamic advice in response to their changes in BGL.

**Table 4:** Applications used by participants and self-management tasks supported by each app [4]

Application	Blood glucose	Exercise	Diet	Medication	Blood pressure	Weight	Self-management education
Dario	Y	Y	Y	Y	N	Y	Y
Fitbit	N	Y	Y	N	N	Y	N
Accu-Chek	Y	Y	Y	Y	Y	Y	N
My Fitness Pal	N	Y	Y	N	N	Y	Y
CronoMeter	Y	Y	Y	N	Y	Y	N
Map My Walk	N	Y	Y	N	N	N	N
BG Star	Y	N	N	Y	N	N	N
MedAdvisor	N	N	N	Y	N	N	Y
Diabetes Journal	Y	Y	Y	Y	Y	Y	N
Glucose Buddy	Y	Y	Y	Y	N	N	N

Most participants responded positively to the suggestion of tailored educational text messages. Text messages represent a relatively novel approach to address management non-adherence and health beliefs [45] and have been demonstrated to improve self-reported adherence to treatment regimes [10, 33]. Participants indicated that weekly would be an appropriate time interval to receive such messages. Future studies could elucidate ways to individualise messages using information stored by diabetes apps.

Several suggestions for features that monitor and track health information were also made, such as incorporating additional monitoring features including blood pressure, weight and activity tracking. A cross-sectional survey of current use of diabetes apps in Australia found that people with T2DM were likely to use multiple functions in apps to support self-management behaviours, with the most used features being blood sugar monitoring and activity and weight tracking [12]. Importantly, El-Gayer and Brzan suggest that these features have the capacity to improve people’s self-management of diabetes [4, 9]. Furthermore, Anderson et al. [5], in alignment with the MARS theme of engagement, found apps that can sustain positive behaviours were more likely to be used on a continual basis. The inclusion of additional features is also important as participants noted their T2DM to be accompanied by comorbidities, which they also considered health priorities. A recent systematic review and meta-analysis have shown that for applications to have a significant effect on HbA1c readings, more than 2 features must be available, particularly including feedback in response to blood glucose trends, which is consistent with the preferences of the participants in our study [36].

### Strengths and limitations

The recruitment process for this study was largely implemented through social media. This may have resulted in an oversaturation of self-selecting, technology literate individuals; however, we were able to identify 14 non-app users in the study. We only included rural participants who may have different needs than their urban counterparts in terms of internet access and transport, although the remainder of our findings would apply to both urban and rural populations. The majority of participants, however, were less than 60 min from their GP. Voices of people living further away from services are therefore missing from our study. These may be an important group in terms of our research questions driving this study because accessibility of healthcare may change participant engagement with apps. We also did not keep a log of how long participants used the apps, which may have provided additional insight into sustainability of app usage. A further limitation was the use of deductive content analysis. As this is a relatively new area of research, an inductive analysis approach may have allowed more novel findings to be included in the data analysis.

There are a variety of items we did not explore but could be considered for future research such as: what motivates participants to install diabetes apps, how do people chose to install any of the apps, patients’ awareness about apps that breach data privacy policies and erroneous insulin dose calculator apps.

A strength of the study was the use of validated theoretical constructs to conceptualise the study. The present study is to our knowledge the 1st study that uses the interview guide developed by Anderson et al. [5], based on the three frameworks, in relation to T2DM. Another strength was that the person with diabetes perspective of self-management using mobile phone applications was sought, a previously identified limitation [5]. Furthermore, this study is the first qualitative study to report the attitudes and experiences of individuals living in rural Australia regarding app use in T2DM, where issues of healthcare access may increase the importance of self-management strategies. Further research could explore the opinions of healthcare professionals to elicit more in-depth understanding of app use to improve T2DM self-management [29, 46].

## Conclusions

Features perceived as useful or facilitated use included the visual representation of trends, encouragement of self-motivation, convenience and user-friendly designs. Important barriers included a lack of awareness and prior consideration of apps in healthcare, inadequate internet access in rural areas, and technological and health literacy. A notable conclusion is the importance of healthcare professionals being aware of apps as a self-management option and being involved in their use to facilitate improved patient outcomes and education. The findings may guide app developers in improving app design and usability. Given self-management is a significant factor in glycaemic control, these findings are significant for GPs, nurse practitioners and allied health professionals who may integrate apps into a holistic management plan which considers strategies outside the clinical environment. Further research is needed to examine the perspective of health professionals in recommending app usage.

## Data Availability

All data generated or analysed during this study are included in this published article and in Appendix.

## Appendix

See Tables 4, 5 and 6.

**Table 5:** Interview guide app users. Frameworks adapted from tool used by Anderson et al. [5]

Question	Elaboration questions	Theory, study, construct
Your gender?		Acceptance factors of mobile apps—sociodemographics
What age bracket do you fit into?	18–25; 26–35, 36–45, 46–55, > 55	
What is your occupation?		
What is your highest level of education?	Year 10, Year 12, TAFE, University	
Where do you currently live?	Is it classified as regional, remote or very remote?	
How far away are you from your GP and endocrinologist?	How long does it take you to get there?	
How frequently are you supposed to attend appointments?		
How difficult is it for you to attend all of your required appointments?	Do you ever miss any because it is too hard?	
How is your diabetes currently managed?	Lifestyle interventions, medication or insulin	Acceptance factors of mobile apps—current state of health
Do you currently use an app or did you use one in the past?		
(if they previously used an app) Why did you stop using the app?	Were there any particular things that lead you to stop using the app?	Acceptance factors of mobile apps—reasons against using smartphones, tablets, and apps
Please tell me about how you use your health app	How did you set it up? What problems do you recall in setting it up? (Prompts: user interface, prompts, permissions, language used)	Usability risk level evaluation
For approximately how long have you used (did you use) this app?	How often do/did you use it? (If discontinued) Why did you stop using the app?	Usability risk level evaluation
On which platform do/did you use this app?	Iphone, Ipad, android phone, android tablet	Usability risk level evaluation; design evaluation-leverage technology familiar to clients
What do/did you like about this app?	Does/did the app fulfil your needs? Why or why not? Do/did you enjoy sessions with your health app? How is/was working with your app satisfying? Is/was your health app worth recommending to others?	TAM—usefulness Mobile App Rating Scale
How easy is/was using your app?	What makes/made the app information clear and understandable? How do/did you find the font size and representation? How do/did you add remarks to your readings?	TAM—ease of use Acceptance factors of mobile apps—perceived ease of use
Have you sometimes not known (did you sometimes not know) what to do next with your app?	Are/were there any parts of the app you don’t use, because they’re complicated? What app features do/did you find unreasonable? Do/did you sometimes wonder if you’re using the app the right way? Who do/would/did you turn to for help using the app (prompts: family, friends, or online forum)?	Acceptance factors of mobile apps—technological literacy
Have you found any ‘bugs’ in your health app, or things it can’t do?	If the app crashes or freezes (crashed or froze), is/was it easy to restart? Have you ever given up due to technical glitches? Have you ever contacted the company about any technical glitches?	Acceptance factors of mobile apps—limitations of the app
How much sight, sound and tactile stimulation do/did you get from your health app?	(Prompts: graphs, things that flash up, reminders about personal targets, warnings, sound effects/reminders, vibration alerts)	Mobile App Rating Scale—engagement
What customization features would you like to see in your health app?		Mobile App Rating Scale—engagement, aesthetics
What is your view of information stored on the cloud?	Do you have concerns about privacy?	Acceptance factors of mobile apps—perceived data security
Does/did your doctor (or other main health care provider) know you have used this app?	(If yes) How would you describe his/her reaction? Are you encouraged by a health professional (pharmacist, general practitioner) to self-reflect on your chronic condition?	TAM—social influence/subject norms
What medical or technical jargon have you seen in your app which you don’t understand?		Design and evaluation guidelines—leverage technology familiar to clients
Does your app use technology you are already familiar with?	Are the dialogue boxes and input fields similar to what you are used to?	Design and evaluation guidelines—leverage technology familiar to clients
What features of your app do you think conflict with each other?	For example: inconsistent short cuts, conflicting educational information	Usability risk level evaluation
Are you satisfied with the time taken to perform tasks on your app?	(Prompts: time to display graphs, time to synchronize information, Are you able to upload data from your blood glucose measuring device?)	Usability risk level evaluation
Do you think that using the app has allowed you to better manage your diabetes?	Prompts: Improved sugar levels, improved medication compliance, encouraged more physical activity, healthy eating, etc.	Mobile App Rating Scale—subjective quality
What (if any) educational features does your current app provide?	Prompt: Do you find it easy to access reliable diabetes information? Where do you go for this information?	Acceptance factors of mobile apps—features and design of a useful app; Mobile App Rating Scale—information quality
What type of information would you be seeking from a mobile phone application?	Prompt – your medication, recipes, nutrition information, general info about diabetes, stress and psychological health What diabetes issues do you think are important to have information on? (can prompt- diet, foot care, hypoglycaemia, hyperglycaemia)	Usability risk level evaluation—interest in new technologies for diabetes treatment and current usage
What part of your diabetes do you struggle with managing the most?	Do you find it hard to find personalized, relevant information?	Mobile App Rating Scale—information targeted
How do you currently access information if you want to educate yourself?	What are the issues you find with current diabetes education platforms?	Usability risk level evaluation—interest in new technologies for diabetes treatment and current usage; design evaluation—leverage technology familiar to clients
What form of information would you find most useful?	Prompts: videos, reading articles, talking to others What are the perceived benefits and barriers to using these different forms	Mobile App Rating Scale—information targeted; design evaluation—leverage technology familiar to clients
Do you think receiving daily text messages or emails with reliable diabetes information would be useful for you?	If not, how often would you like to receive information?	Acceptance factors of mobile apps—features and design of a useful app; design evaluation—leverage technology familiar to clients

**Table 6:** Interview guide non-app users. Frameworks adapted from tool used by Anderson et al. [5]

Question	Elaboration questions	Theory, study, construct
Your gender?		Acceptance factors of mobile apps—sociodemographics
What age bracket do you fit into?	18–25; 26–35, 36–45, 46–55, > 55	
What is your occupation?		
What is your highest level of education?	Year 10, Year 12, TAFE, University	
Where do you currently live?	Is it classified as regional, remote or very remote?	
How far away are you from your GP and endocrinologist?	How long does it take you to get there?	
How frequently are you supposed to attend appointments?		
How difficult is it for you to attend all of your required appointments?	Do you ever miss any because it is too hard?	
How is your diabetes currently managed?	Lifestyle interventions, medication or insulin	Acceptance factors of mobile apps—current state of health
Do you currently or have you ever used a mobile phone app to help manage your diabetes?	If yes than refer to other interview guide for app users.	
What are your main reasons for not using a diabetes app?	Do you have concerns about using a mobile phone app for health purposes?	Acceptance factors of mobile apps—reasons against using smartphones, tablets, and apps
Would you ever consider using a mobile phone app to help manage your diabetes?		Usability risk level evaluation
If you were to use an app, what features would you want in the app to make it useful to you?	May prompt: Education, reminders to check blood sugars, recipes/diet info, exercise	TAM—usefulness
How does your current practitioner encourage you to monitor your own health?	Has your practitioner ever mentioned/recommended a health app to you?	TAM—social influence/subject norms
Do you regularly record blood sugar levels?	What methods do you use to keep track of your sugar levels?	Acceptance factors of mobile apps—features and design of a useful app
What techniques, if any, do you use to ensure you always remember to take your medications?	Do you ever forget? Would an app that reminded you to take your medication be helpful?	Acceptance factors of mobile apps—features and design of a useful app
Do you have any alternative methods to motivate yourself to eat healthy and exercise regularly?	If so, please describe them.	Acceptance factors of mobile apps—features and design of a useful app
What type of information would you be seeking from a mobile phone application?	Prompt—your medication, recipes, nutrition information, general info about diabetes, stress and psychological health What diabetes issues do you think are important to have information on? (can prompt- diet, foot care, hypoglycaemia, hyperglycaemia)	Mobile App Rating Scale—information targeted
What part of your diabetes do you struggle with managing the most?	Do you find it hard to find personalized, relevant information?	Mobile App Rating Scale—information targeted
How do you currently access information if you want to educate yourself?	What are the issues you find with current diabetes education platforms?	Usability risk level evaluation—interest in new technologies for diabetes treatment and current usage; design evaluation—leverage technology familiar to clients
What form of information would you find most useful?	Prompts: videos, reading articles, talking to others What are the perceived benefits and barriers to using these different forms	Acceptance factors of mobile apps—features and design of a useful app; design evaluation—leverage technology familiar to clients
Do you think receiving daily text messages or emails with reliable diabetes information would be useful for you?	If not, how often would you like to receive information?	Acceptance factors of mobile apps—features and design of a useful app; design evaluation—leverage technology familiar to clients

## Abbreviations

T2DM: Type Two Diabetes Mellitus
Apps: mobile phone applications
BGL: blood glucose level(s)
GP: general practitioner
MARS: Mobile Application Rating Scale
TAM: Technology Acceptance Model
HITAM: Health Information Technology Acceptance Model
